# A Genomic Duplication is Associated with Ectopic Eomesodermin Expression in the Embryonic Chicken Comb and Two Duplex-comb Phenotypes

**DOI:** 10.1371/journal.pgen.1004947

**Published:** 2015-03-19

**Authors:** Ben Dorshorst, Mohammad Harun-Or-Rashid, Alireza Jian Bagherpoor, Carl-Johan Rubin, Chris Ashwell, David Gourichon, Michèle Tixier-Boichard, Finn Hallböök, Leif Andersson

**Affiliations:** 1 Science for Life Laboratory, Dept. of Medical Biochemistry and Microbiology, Uppsala University, Uppsala, Sweden; 2 Dept. of Animal and Poultry Sciences, Virginia Tech, Blacksburg, Virginia, United States of America; 3 Dept. of Neuroscience, Uppsala University, Uppsala, Sweden; 4 Prestage Dept. of Poultry Science, North Carolina State University, Raleigh, North Carolina, United States of America; 5 INRA, UE 1295 PEAT Pôle d'Expérimentation Avicole de Tours, Nouzilly, France; 6 INRA, AgroParisTech, UMR1313 Animal Genetics and Integrative Biology, Jouy-en-Josas, France; 7 Science for Life Laboratory, Dept. of Animal Breeding and Genetics, Swedish University of Agricultural Sciences, Uppsala, Sweden; University of Bern, SWITZERLAND

## Abstract

*Duplex-comb* (*D*) is one of three major loci affecting comb morphology in the domestic chicken. Here we show that the two *Duplex-comb* alleles, V-shaped (D*V) and Buttercup (D*C), are both associated with a 20 Kb tandem duplication containing several conserved putative regulatory elements located 200 Kb upstream of the *eomesodermin* gene (*EOMES*). EOMES is a T-box transcription factor that is involved in mesoderm specification during gastrulation. In D*V and D*C chicken embryos we find that EOMES is ectopically expressed in the ectoderm of the comb-developing region as compared to wild-type embryos. The confinement of the ectopic expression of EOMES to the ectoderm is in stark contrast to the causal mechanisms underlying the two other major comb loci in the chicken (*Rose-comb* and *Pea-comb*) in which the transcription factors MNR2 and SOX5 are ectopically expressed strictly in the mesenchyme. Interestingly, the causal mutations of all three major comb loci in the chicken are now known to be composed of large-scale structural genomic variants that each result in ectopic expression of transcription factors. The *Duplex-comb* locus also illustrates the evolution of alleles in domestic animals, which means that alleles evolve by the accumulation of two or more consecutive mutations affecting the phenotype. We do not yet know whether the *V-shaped* or *Buttercup* allele correspond to the second mutation that occurred on the haplotype of the original duplication event.

## Author Summary

There are three major variant comb types found in the domestic chicken; Rose-comb, Pea-comb and Duplex-comb. Within the Duplex-comb there are two distinct types, V-shaped and Buttercup. Previous experiments have shown that these two Duplex-comb types represent different alleles at a single locus. We have mapped the location of the *Duplex-comb* locus and identified a 20 Kb duplication that is present only in chickens that have a Duplex-comb phenotype. The 20 Kb duplication is located 200 Kb upstream of *EOMES*, a gene that was found to be abnormally expressed in the comb-developing region of V-shaped and Buttercup comb chicken embryos. This suggests that the 20 Kb duplication contains regulatory elements affecting *EOMES* expression. These findings complete our characterization of the genetic basis of the three major comb loci in the chicken, all of which are caused by large-scale structural genomic variants that drive ectopic expression of transcription factors in the comb region during chicken embryo development.

## Introduction

In the domestic chicken (*Gallus domesticus*) the comb serves as a sexual ornament and the size of the comb is associated with mate choice in both sexes as well as fecundity in females [1,2]. The vast majority of chicken populations used for commercial meat and egg production around the world are fixed for the wild-type single comb phenotype. However, there are three major comb loci found in non-commercial chicken breeds which are primarily used for exhibition purposes; Rose-comb, Pea-comb and Duplex-comb. The causal mutations for Rose-comb and Pea-comb have recently been identified, both corresponding to structural genomic variants that drive ectopic expression of transcription factors in the developing comb region of the chicken embryo [3–5].

**Fig 1:**
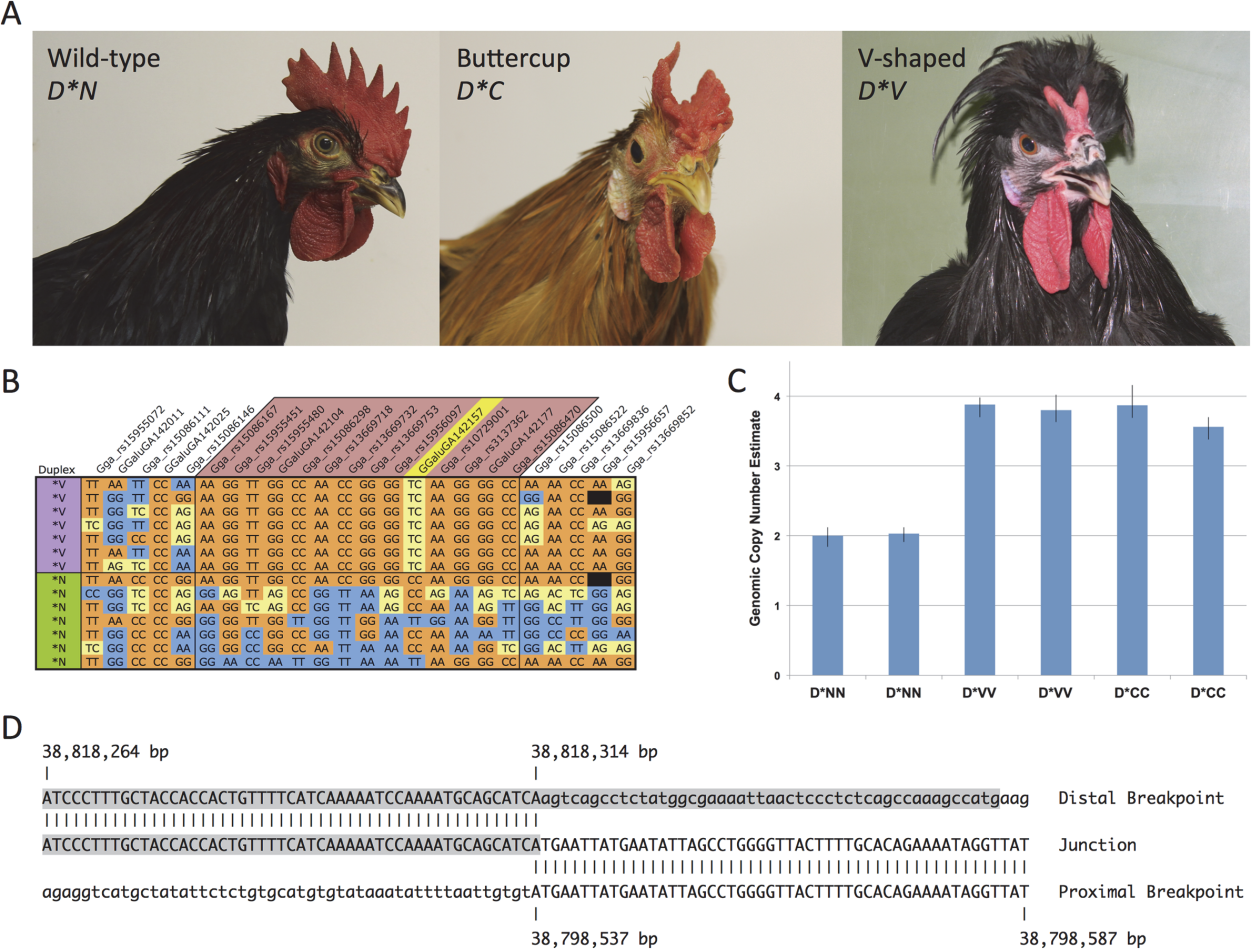
The Duplex-comb phenotypes V-shaped and Buttercup are both associated with a novel 20 Kb duplication on chicken chromosome 2. (A) Phenotypes associated with the *Duplex-comb* locus V-shaped (D*V), Buttercup (D*C) and wild-type (D*N) single comb alleles. (B) A 381 Kb haplotype is IBD in D*V individuals, SNP names in the region marked in red. Yellow shading represents heterozygous genotypes with blue/orange representing reciprocal homozygous genotypes. Genotype data is from the 60K SNP chip and color coded according to genotype with missing data shown in black. A single SNP GGaluGA142157 at 38,806,246 bp (marked in yellow) is heterozygous in all D*V individuals, suggestive of a duplication. A broader view of haplotypes in more individuals is available in S1 Fig. (C) A TaqMan assay was used to investigate the genomic copy number of the putative duplicated region, showing that a duplication is present in both D*V and D*C individuals. Each bar represents a single individual. Error bars represent the minimum and maximum estimated copy number as calculated from technical replicates of each sample by Copy Caller software (ABI). (D) The *Duplex-comb* alleles are associated with a tandem duplication with no sequence homology at the junction point. The middle line shows the sequence at the junction point with vertical dashes indicating sequence alignment to the 5’ (bottom line) and 3’ (top line) boundaries of the duplicated region. A LTR element is shaded in gray.

The *Duplex-comb* locus harbors three alleles, Buttercup (D*C), V-shaped (D*V) and wild-type or normal (D*N). Chickens that are wild-type at the *Rose-comb*, *Pea-comb* and *Duplex-comb* loci have the single comb phenotype. D*C corresponds to a cup shaped comb arising from a single central blade ringed with individual points along the perimeter of the cup (Fig. 1A). This phenotype is somewhat rare, being found in the Sicilian Buttercup, Caumont and Augsburger chicken breeds. Chickens that appear to have the Buttercup comb phenotype were described by the naturalist Ulisse Aldrovandi in 1600 [6,7] and are thought to originate from North Africa, possibly being the progenitors of the Sicilian Buttercup breed.

D*V corresponds to a two-pronged horn or V-shaped comb that is restricted to the posterior portion of the comb developing region (Fig. 1A). The V-shaped comb is found in many breeds from around the world such as the Crevecoeur, Houdan, La Fleche, Merlerault, Padova, Polish, Spitzhauben and Sultan. Both comb types can vary slightly in shape and size between breeds and strains due to differences in genetic background, with D*C occasionally resembling two distinct single (wild-type) combs split down the midline. Previous experiments have demonstrated that the V-shaped and Buttercup phenotypes are inherited as determined by alleles at a single locus [8]. In these experiments D*V was completely dominant over D*C. Both mutant alleles were incompletely dominant over D*N. D*V was completely penetrant in both sexes while D*C was incompletely penetrant (68%) in females [8]. From these experiments it was suggested that D*C represents a comb doubling effect while D*V causes doubling and reduction of comb size.

Here we show that both Duplex-comb phenotypes are associated with a 20 Kb tandem duplication and ectopic expression of EOMES, a transcription factor with a known role in mesoderm specification in the developing embryo [9].

## Results

### Identification of the genomic region associated with Duplex-comb

We previously mapped D*V to the 37.3–39.8 Mb region of *Gallus gallus* autosome 2 (GGA2) in a backcross population [10]. Subsequent fine mapping with additional markers identified a region of maximum association with D*V as between markers rs15086167 and rs14167302, corresponding to GGA2:38,554,221–39,229,442 bp.

Genotyping of a diverse breed panel (D*V, n = 7; D*N, n = 64) on the 60K Chicken iSelect chip [11] (Illumina) revealed an identical by descent (IBD) haplotype located between markers rs15086146 and rs15086500, corresponding to GGA2:38,528,939–38,910,305 bp and consistent with other reports [12]. A single SNP within the IBD haplotype was observed to be heterozygous in all D*V individuals, (GGaluGA142157 at 38,806,246 bp), suggestive of a duplication fixed for alternative SNP variant alleles (Figs. 1B and S1).

Copy number of the IBD region was explored using SYBR Green qPCR analysis with genomic DNA as template. Iterative rounds of qPCR analysis of copy number analysis ultimately defined the approximate boundaries of a putative 2-fold duplicated region in D*V individuals. Genomic copy number analysis using a TaqMan assay was in agreement with the SYBR Green assays and confirmed the presence of a duplication in both D*V and D*C individuals as compared to D*N (Fig. 1C).

Successful amplification and sequencing across the duplication junction point revealed this to be a tandem duplication of a ∼20 Kb segment spanning from 38,798,537 to 38,818,314 bp. Analysis of the duplication junction sequences revealed only a single base pair of overlap and no other sequence micro-homologies (Fig. 1D). The 38,818,314 bp duplication junction point is within a LTR element as annotated by the UCSC Genome Browser.

A diagnostic PCR test was designed to amplify across the duplication junction point and used to screen a diverse breed panel representing the three known alleles at the *Duplex-comb* locus. All D*N homozygotes (n = 44) were wild-type for the duplication junction point while all V-shaped (n = 48) and Buttercup (n = 35) individuals had at least one copy of the duplication (Table 1). This PCR test cannot distinguish between heterozygotes and homozygotes for the duplication as no wild-type sequences are disrupted in this tandem duplication.

**Table 1:** A PCR-based diagnostic test reveals complete association of a duplication junction point with the Duplex-comb phenotypes in chickens.

Breed	Duplication	Wild-type
D*V (V-shaped)		
Crevecoeur	2	0
Houdan	26	0
Padova	7	0
Polish	7	0
Spitzhauben	2	0
Sultan	4	0
D*C (Buttercup)		
Sicilian Buttercup	29	0
Caumont	6	0
D*N (Wild-type)		
Araucana	0	2
Ayam Cemani	0	7
Blue Egg	0	2
Brahma	0	4
Campine	0	2
Cochin	0	3
Dorking	0	2
Faverolle	0	2
Hamburg	0	3
Junglefowl	0	1
Leghorn	0	1
Mixed Layer	0	2
Orpington	0	1
Plymouth Rock	0	3
Sebright	0	1
Silkie	0	4
Sussex	0	2
Wyandotte	0	2
***Total***	83	44

Samples from the Caumont, Crevecoeur and Padova breeds were supplied by INRA, some of which were part of the AvianDiv Project [38]. All other samples were collected in the USA except for Sicilian Buttercup and Houdan, which were collected in both countries.

### Whole genome sequencing for the characterization of the D*V and D*C haplotypes

Three chickens, each representing one of the three alleles at the *Duplex-comb* locus, were selected for whole genome sequencing to search for mutations other than the 20 Kb duplication that could be responsible for the difference between the V-shaped and Buttercup comb phenotypes. The average depth of sequence coverage for each bird was in the range 24x to 34x, which gives a high power for SNP detection at most sites. The D*V (White Crested Black Polish, USA) and D*C (Sicilian Buttercup, Italy) individuals were from breeds with standardized V-shaped and Buttercup comb phenotypes and were tested with the TaqMan copy number assay to verify homozygosity before whole genome sequencing. The D*N (Single Comb Dark Brown Leghorn, USA) individual was selected due to sharing an identical haplotype as the D*V individual based on the 60K SNP chip genotype data except for the D*V associated heterozygous SNP and 20 Kb duplication.

**Fig 2:**
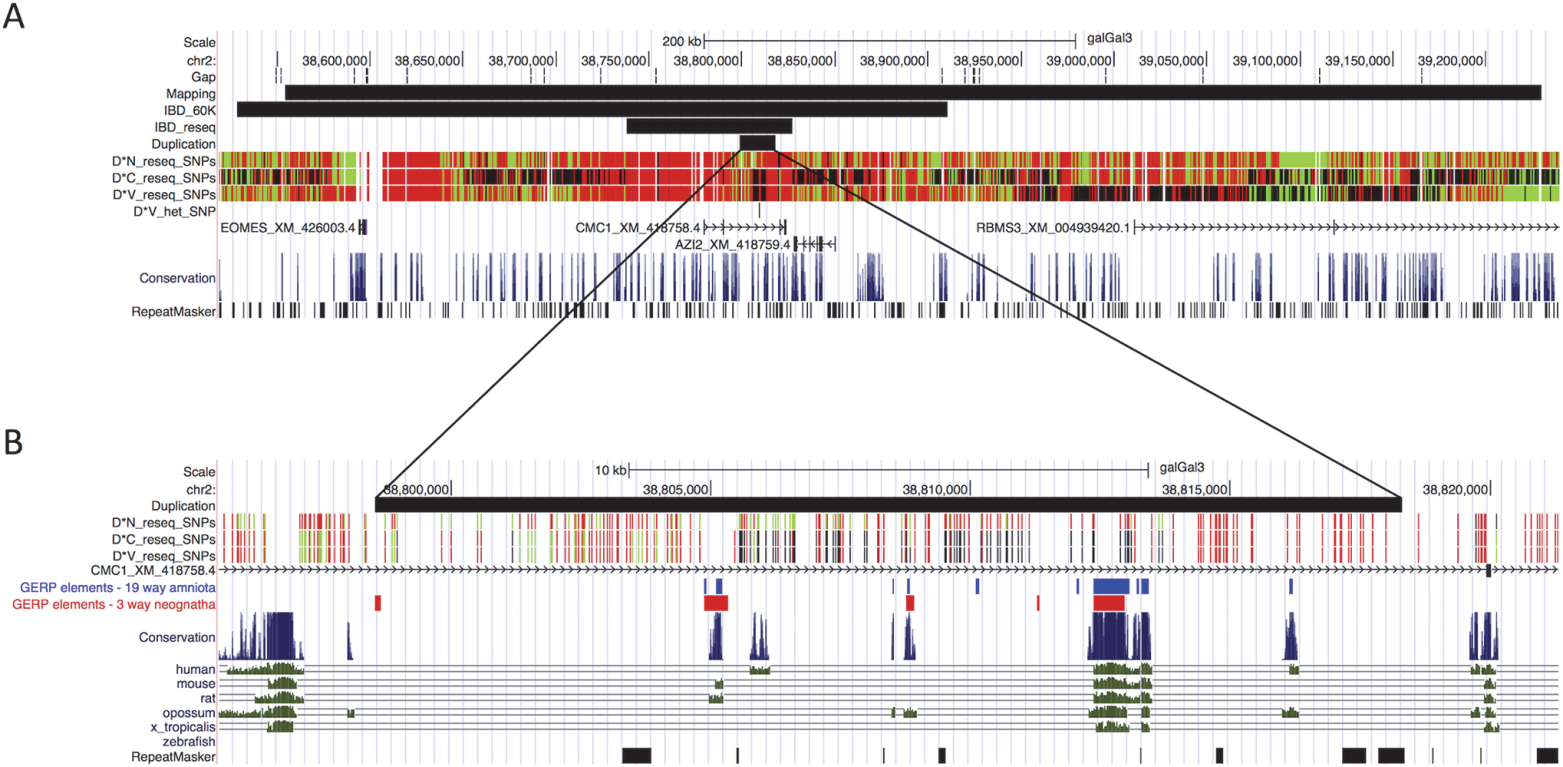
Characterization of the *Duplex-comb* locus by genetic mapping and whole genome sequencing. (A) The genomic region to which the *Duplex-comb* locus was mapped to; adapted from the UCSC Genome Browser. Black bars represent regions identified from the backcross mapping population, IBD data from the 60K SNP chip, IBD data from whole genome sequencing and the 20 Kb duplication. SNPs identified from whole genome sequencing are shown in Green, Black and Red corresponding to homozygous reference allele, heterozygous, and homozygous variant allele, respectively. Although not currently annotated in the galGal3 genome build four genes are predicted in this region: *EOMES* (XM_426003.4), *CMC1* (XM_418758.4), *AZI2* (XM_418759.4) and *RBMS3* (XM_004939420.1). The 20 Kb duplicated region is located ∼200 Kb upstream of *EOMES*. (B) Several regions within the 20 Kb duplication show elevated conservation and Genomic Evolutionary Rate Profiling (GERP) scores for 19 amniota (blue) and 3 neognath (red) species.

The largest region for which D*C and D*V individuals were IBD was 89 Kb in size (38,738,016–38,827,468 bp) which includes the entire 20 Kb duplicated region (Fig. 2A, IBD_reseq track). We identified 6 and 17 paired-end reads that spanned the duplication junctions in D*V and D*C individuals, again confirming the exact duplication breakpoints. We then used the sequencing data to explore if there were any other sequence variants that showed a perfect concordance with D*V and D*C like the 20 Kb duplication. Stringent SNP calling revealed only one high-quality SNP, at position 38,797,948 bp, within the IBD region that showed this pattern and that were not found in other chicken populations with the single comb phenotype [13]. This SNP did not occur at an evolutionary conserved site.

To identify one or more mutations that distinguish the two mutant alleles we first searched for SNPs within the duplicated region. There were 182 SNPs detected between all three sequenced individuals, with 181 SNPs having identical genotypes in D*C and D*V individuals (Fig. 2B). The one remaining SNP at 38,808,838 bp was heterozygous G/A in the D*V individual and homozygous reference (G) in the D*C and D*N individuals chosen for sequencing. Further screening showed that this SNP was homozygous reference (G) in 23 of 32 additional D*V individuals from four different breeds and was never found to be homozygous for the mutant allele. This indicates that the 38,808,838 bp SNP is not causally associated with D*V, but instead has evolved in some D*V populations in one of the two copies of the 20 Kb duplicated region. There were no other high-quality SNPs within the 89 Kb IBD region identified from the sequencing data for which the V-shaped and Buttercup individuals were homozygous for alternative alleles.

There were 66 SNPs within the duplicated region that were called as heterozygous in both the D*C and D*V individuals, indicative of the two copies of the duplicated region composing a single haplotype, each copy carrying different sequence variants at 66 SNP positions. Thus, the nucleotide divergence between the two copies is about 0.3%, i.e. three times higher than the average nucleotide diversity in the human genome and close to the average nucleotide diversity of 0.5% in the chicken genome [14]. This implies a scenario where two different haplotypes contributed to the tandem duplication and the majority of the sequence differences are expected to represent sequence differences between the two ancestral haplotypes. This interpretation is consistent with the observation that most SNPs showing sequence differences between the two copies, such as GGaluGA142157 at 38,806,246 bp, also segregated among wild-type chromosomes (Fig. 1B).

There are several regions within the 20 Kb duplication that exhibit elevated conservation scores according to the UCSC genome browser and Genomic Evolutionary Rate Profiling (GERP) [15] (Fig. 2B), representing putative regulatory elements.

### Morphology

The chicken comb originates from a region on the upper beak, posterior to the fronto-nasal facial process and is first visible as a narrow midline ridge, at embryonic day 6–7 (E6–7). The wild-type single comb has one row of papillae that are formed from local mesenchyme condensations along the initial comb-ridge and they present the beginnings of the comb serrations (S2A and B Fig.). The V-shaped (D*V) and Buttercup (D*C) combs are initially formed by a split of this single comb anlage. The split occurs at a variable posterior position in the V-shaped comb ridge with a reduction of the anterior portion of the comb while in the Buttercup the whole ridge is split. The posterior part of the split ridge in Buttercup is often fused as seen in S2E Fig.. The appearance of the developing nostrils is also affected in the duplex phenotype.

### Expression analysis reveals ectopic expression of EOMES in comb tissue from Duplex-combed individuals

**Fig 3:**
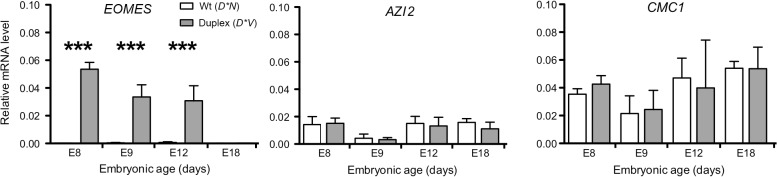
*EOMES* expression is increased in D*V embryonic comb tissue. Results of qRT-PCR analysis demonstrating increased *EOMES* expression in D*V embryonic comb tissue whereas *CMC1* and *AZI2*, located nearby the duplicated region, do not show any significant change in expression. Bar graphs mean ±sem, ANOVA, *P<0.05, **P<0.001, ***P<0.0001.

The expression pattern of candidate genes located in the proximity of the 20 Kb duplication was investigated using quantitative reverse transcription-PCR (qRT-PCR) in samples of comb tissue from developing chicken embryos. The duplication is located within an intron of *CMC1* encoding COX assembly mitochondrial protein homolog (*S*. *cerevisiae*) as assessed by aligning the predicted *CMC1* sequence (XM_418758.4) to the chicken genome via BLAT in the UCSC genome browser. 5-azacytidine induced 2 (*AZI2*) is the nearest gene on the 3’ side of the duplication and eomesodermin (*EOMES*) is the nearest gene on the 5’ side. *CMC1* and *AZI2* were both expressed in comb tissue but did not show any significant difference in expression between genotypes. In contrast, *EOMES* showed a dramatic expression difference between genotypes and was more highly expressed at E8, E9 and E12 in D*V embryonic comb as compared to D*N, while it was not expressed in any genotype at E18 (Fig. 3).

**Fig 4:**
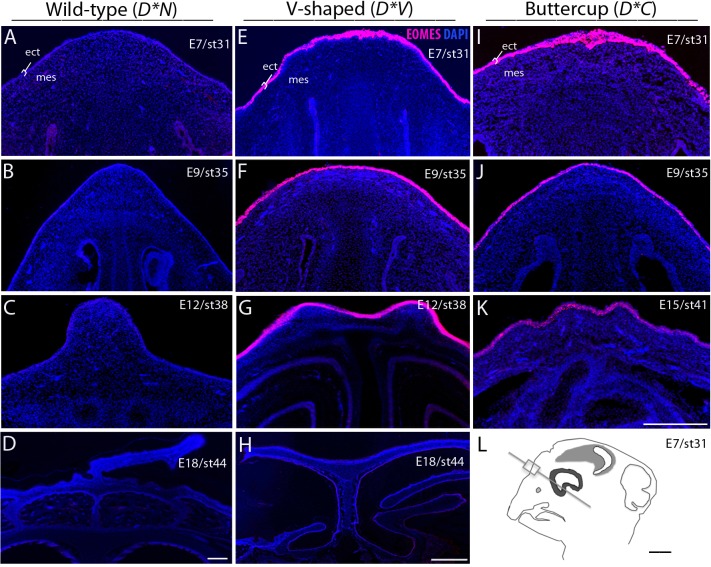
Ectopic expression of EOMES in the ectoderm of D*V and D*C embryonic chicken comb tissue. A-D: Immunohistochemical labeling did not detect any expression of EOMES in either the mesenchyme or ectoderm of the D*N developing comb region. E-K: EOMES is expressed in the ectoderm of both D*V and D*C embryos as early as embryonic day 7 (E7) and continuing through E12/E15. All genotypes showed similar patterns of expression of EOMES in the brain, which was used as a positive control for the antibody specificity. By E12 and E15 a change in tissue morphology is readily apparent in both D*V and D*C embryos. ect; ectoderm, mes; dermal mesenchyme. Scale bar in K is 200 μm and applies to A-C, E-G, I and J. Bars in D and H are 100 μm and L is 1 mm.

The spatial distribution of EOMES expression in the developing comb region of the chicken embryo was investigated using immunohistochemistry. In D*N embryos no EOMES expression was detected from E7-E18 in the ectoderm or mesenchyme of the comb region. Both D*V and D*C embryos showed clear expression of EOMES in the ectoderm of the comb region already at E7 and continuing through E12/E15 (Fig. 4E-G, I-K). The expression of EOMES was limited to the ectoderm of the developing comb region at all stages analyzed in D*V and D*C embryos and could not be detected by E18 (Fig. 4H).

## Discussion

The *Duplex-comb* locus was originally described as having two mutant alleles [8] and being linked to the polydactyly locus [16], which was subsequently mapped to GGA2 [10,17–19]. Through successive rounds of linkage mapping and IBD haplotype analysis using different chicken breeds we have identified an 89 Kb region of GGA2 as encompassing the *Duplex-comb* locus. This region contains a 20 Kb tandem duplication that is only found in chicken breeds that have a Duplex-comb phenotype when screened on a diverse breed panel. Sequence analysis of the duplicated region identified only a single base pair difference within the 20 Kb duplication between the two mutant alleles, however this variant was subsequently excluded as the causal difference between D*C and D*V alleles after finding many D*V individuals that were homozygous reference. The 20 Kb duplication contains several putative conserved regulatory elements (Fig. 2B) that is likely driving the ectopic expression of the downstream transcription factor EOMES in the developing chicken comb region in Duplex comb individuals.

The phenotypic diversity of the chicken comb is primarily governed by a small number of loci with large effects that determine the overall morphology of the comb during embryonic development; the *Rose-comb*, *Pea-comb* and *Duplex-comb* loci. The *Rose-comb* and *Pea-comb* loci are notable in being the first example of classical genetic epistasis, giving rise to the Walnut comb phenotype when mutant alleles are present at both loci [20]. The *Pea-comb* mutant allele has recently been described as corresponding to a copy number expansion in an intron of *SOX5*, resulting in ectopic expression of this transcription factor in the mesenchyme of the developing comb region of the chicken embryo [5]. The *Rose-comb* mutant allele was also recently characterized, corresponding to a 7 Mb inversion that leads to ectopic expression of the transcription factor MNR2 in the mesenchyme of the developing comb region of the chicken embryo [4]. This overlapping spatial and temporal domain of ectopic expression of SOX5 and MNR2 is a clear demonstration of how the epistasis between *Rose-comb* and *Pea-comb* loci is derived at the cellular level through the combined action of two transcription factors [4]. Here we show that the last major comb locus in the chicken to be characterized at the molecular level also corresponds to a structural variant in the chicken genome that results in ectopic expression of a transcription factor. However, while Rose-comb and Pea-comb phenotypes are driven by ectopic expression of SOX5 and MNR2 in the mesenchyme of the developing comb region, we show that the Duplex-comb phenotype is mediated by ectopic expression of EOMES confined to the ectoderm.

Eomesodermin (*EOMES*) is a T-box transcription factor that is involved in mesoderm specification during gastrulation as shown in zebrafish, chicken and mouse. EOMES is expressed in the extraembryonic tissues of the chicken and the mouse as well as the primitive streak, forebrain region and genital ridge [9,21]. However, expression of EOMES in primordial germ cells is only found in the chicken [21]. Investigation of four upstream and one downstream putative cis-regulatory element (CRE) of mouse *EOMES* indicated that different regulatory mechanisms between mouse and chicken were likely responsible for EOMES expression in extraembryonic tissues while a single CRE located ∼150 Kb upstream drove expression in the brain in both chicken and mouse [21]. The 20 Kb duplication overlaps several regions of elevated sequence conservation and lies approximately 200 Kb upstream of *EOMES* in the chicken, suggesting that the duplicated region contains CREs and that an altered dosage of these elements causes perturbed regulation of *EOMES* expression.

Using qRT-PCR we show that *EOMES* is upregulated in the comb region of D*V embryos as early as embryonic day 8 as compared to D*N embryos. There was no difference in expression of *CMC1* and *AZI2* (the two other genes located nearest the 20 Kb duplication) between D*V and D*N embryos, suggesting that this mutation involves a CRE specific to *EOMES*, at least in comb tissue. Using IHC we show that EOMES is ectopically expressed in the ectoderm of the developing comb region of D*C and D*V embryos. There was no detectable EOMES expression in this region of the D*N embryo at these stages, suggesting that EOMES does not normally play a role in comb development.

The major comb phenotypes are all caused by mutations that direct expression of transcription factors to the ectoderm or mesenchyme of the comb ridge. The development of the comb as part the chicken naso-facial processes is directly induced and regulated by reciprocal ecto-mesenchymal interactions [22]. Interactions of ectopically expressed transcription factors either in the ectoderm or mesenchyme then cause the similar but not identical comb phenotypes. The exact regional and temporal expression of the inductive signals or their receptors is instrumental for the morphogenesis [3]. The D*C phenotype is characterized as a splitting of the comb mass while the D*V phenotype involves both splitting and reduction of comb mass as well as enlargement of the nostrils [6]. We propose that the initial duplication event is the primary driver of ectopic EOMES expression in the ectoderm of the comb region and causes the majority of the comb duplication phenotype. A subsequent and unknown mutation is suspected of further modifying the spatial or temporal expression of EOMES to result in two different Duplex-comb phenotypes, but does so in a manner that escapes the resolution of our IHC experiments.

The mutation that distinguishes the two mutant alleles should be found in a D*C or D*V IBD region. Our initial genotyping data identified a 381 Kb IBD haplotype in D*V individuals (Fig. 1B), however it is uncertain which mutant allele evolved first and we lack similar data for D*C individuals. We restricted our search for a causal mutation to the 89 Kb IBD region since this study shows that this region contains regulatory elements that affect EOMES expression during comb development, but we found no high-quality SNP where the sequenced D*V/D*V and D*C/D*C birds were homozygous for different alleles. A causal mutation could have been overlooked due to a gap or lack of adequate coverage in the sequence data although we had on average high sequence coverage (in the range 24x to 34x); the current assembly of the 89 Kb region contains one gap annotated as comprising about 750 nucleotides. Although prior experiments [8] found that D*C and D*V were alleles of the same locus, it remains unknown how close the mutation differentiating these two alleles lies to the 20 Kb duplication. At present we cannot exclude the possibility that the causal difference between D*V and D*C could be affecting a nearby gene other than *EOMES*.

A common feature of duplicated sequences is that they show copy number variation because nearly identical tandem copies are prone to unequal crossing-over or slippage during replication [23]. We did not detect any such copy number variation and all D*C and D*V chromosomes analyzed in this study appeared to contain only two copies of the duplicated sequence. Furthermore, the two copies of the duplicated sequence showed a 0.3% sequence divergence and were identical between V-shaped and Buttercup chromosomes (except at the SNP distinguishing D*V and D*C sequenced individuals). This implies that the sequence divergence between the two copies is sufficient to suppress unequal crossing-over that may otherwise lead to copy number variation and gene conversion, resulting in homogenization of the tandem copies.

The investigation of genetic mechanisms underlying phenotypic diversity in domestic animals has revealed that structural variation plays a significant role, typically affecting spatio-temporal gene expression patterns through rearrangement of regulatory elements [24]. Examples of such traits are Pea-comb [5], Rose-comb [4], Fibromelanosis [25] and Dark Brown plumage [26] in the chicken; Dominant White in the pig [27]; Greying with age in the horse [28] and Color Sidedness [29] and Polled in cattle [30–32]. Here we add the Duplex-comb locus to this list, highlighting how large-scale genomic mutations appear to often result in very noticeable phenotypic effects that are then easily selected by humans during animal domestication and breeding. The Duplex-comb trait also illustrates another striking feature of genetic diversity in domestic animals, the evolution of alleles [24]. The evolutionary history of domestic animals is sufficiently long to allow the accumulation of two or more causative mutations on the same haplotype. This is the case for instance with Dominant white color in pigs [27], Black spotting in pigs [33] and Rose-comb in chickens [4]. The Duplex-comb locus can now be added to this growing list of examples since the Buttercup and V-shaped alleles share an 89 Kb IBD region including the 20 Kb duplication but differ at a yet unknown position. This illustrates why domestic animals present a valuable model to study the genetic mechanisms and processes that likely underlie phenotypic traits in humans and other species.

## Materials and Methods

### Fine mapping and IBD analysis

A custom GoldenGate BeadXpress panel (Illumina) containing 28 SNPs on GGA2 was used to fine map the D*V mutation in the same backcross population we previously reported [10]. The 60K Chicken iSelect chip [11] (Illumina) was used to genotype a diverse panel of chicken breeds for IBD haplotype analysis. All genome coordinates are relative to the May 2006 WUGSC 2.1/galGal3 assembly.

### Genomic copy number analysis

SYBR Green assays for genomic copy number were performed using SYBR Green PCR Master Mix (ABI) with 800 nM of each primer and 10 ng of DNA in a total volume of 10 μl. Reactions were performed in quadruplicate and data was analyzed using the 2-ΔΔCt method [34], correcting for amplification efficiency as measured by a standard dilution series. TaqMan assays and data analysis for genomic copy number were performed as previously described [25]. A primer/probe set in an exon of *SOX5* was used as a calibrator for both SYBR Green and TaqMan assays. All primer sequences can be found in S1 Table.

### Diagnostic PCR test for duplication junction point

A three primer PCR diagnostic test was developed that amplified over the duplication junction point as well as amplifying a product over one of the duplicated region wild-type sequences. The KAPA2G Robust HotStart PCR system (Kapa Biosystems) was used with 1X KAPA2G GC Buffer, 0.2 mM dNTPs, 1.5 mM MgCl_2_, 200 nM of primers D_5'_F and D_5'_R, 150 nM of primer D_3'_F, 0.4 U of KAPA2G Robust HotStart DNA Polymerase, and 50 ng of DNA in a total volume of 10 μl. A touchdown thermal cycling protocol was used for the diagnostic test of 95°C for 5 min, 16 cycles of 95°C, 68°C (-1.0°C/cycle), and 72°C for 30 s each, followed by 24 cycles of 95°C, 52°C, and 72°C for 30 s each. This test is not capable of differentiating homozygous mutant individuals from heterozygotes.

### Whole genome sequencing

DNA was prepared from blood samples of single individuals representing the D*V (White Crested Black Polish, USA), D*C (Sicilian Buttercup, France) and D*N (Single Comb Dark Brown Leghorn, USA) alleles. The DNA was used to construct paired-end libraries with average insert size of approximately 220 bp and these libraries were subjected to whole genome sequencing using a HiSeq sequencing instrument (Illumina). Sequencing reads (2 x 100bp) were aligned to the chicken reference genome (galgal3) using the Burrows Wheeler Aligner (BWA) [35], revealing average depths of coverage of 24, 25 and 34 for D*V, D*N and D*C, respectively. The aligned reads were subjected to duplicate flagging using Picard Tools (http://picard.sourceforge.net) and to SNP calling using the Genome Analysis Toolkit (GATK) Unified Genotyper version 2.4.9 [36]. Identified raw SNPs were filtered based on GATK best practice variant detection and genotypes with a PHRED genotype quality ≥ 20 were used in subsequent steps. SNP- and genotype calls were compared to SNPs detected in DNA pools from wild- and domestic chickens in a previously published study [13].

### Gene expression analysis

Total RNA was extracted from comb tissue from E8, E9, E12 and E18 D*V (Merlerault, France) and D*N (Geline de Touraine, France) chicken embryos using TRIzol (Invitrogen). RNA was treated with DNase (1 μg/μl) and cDNA was made from 1 μg of RNA using High Capacity RNA-to-cDNA Kit (ABI). The qRT-PCR analysis was performed using CFX96 SyBr Green Supermix (Bio-Rad) with primers designed by using Primer Express v2.0 (ABI), checked for PCR efficiency, linear dynamic range and specificity. The mRNA levels were normalized to β-actin mRNA levels. The use of β-actin for normalization purposes was validated by testing for the most stable mRNA expression of TATA box binding protein, β-actin, ß-2-microglobulin and glyceraldehyde 3-phosphate dehydrogenase over the developmental stages using geNorm [37]. Expression levels were calculated from cycle threshold (Ct) and the 2-ΔΔCt method [34]. The normalized amplification levels of Duplex-comb and single-comb samples relative to the ß-actin amplification levels are shown, and differences were tested by using one-way analysis of variance (ANOVA) followed by Tukey’s range test as indicated in figure legend.

### Immunohistochemistry

Chicken embryo heads from D*V (Merlerault, France), D*C (Caumont, France), and D*N (Geline de Touraine, France) breeds were fixed in 4% paraformaldehyde, pH 7.4 in PBS for one hour at 4°C, transferred to 30% sucrose in PBS overnight at 4°C, frozen in OCT freezing medium and sectioned 10 μm with a cryostat. The sections were washed in PBS and used for immunohistochemistry. Sections were blocked (PBS with 1% fetal calf serum, 0.1% Triton-X, 0.02% Thimerosal) before addition of primary antibodies in blocking solution and incubated overnight at 4°C. The slides were washed three times for 5 min in PBS before incubation secondary antibodies in blocking solution in room temperature for two hours. The slides were washed three times 5 min with PBS before mounting. Primary antibody: TBR2/EOMES (Abcam #ab23345), rabbit polyclonal 1:1000. Secondary antibody: Alexa Fluor 568, rabbit IgG (Invitrogen) was made in donkey. Images from immunohistochemistry were captured using a Zeiss Axioplan2 microscope and AxioVision 4.8 software (Carl Zeiss).

## Supporting Information

S1 Fig Identification of a 381 Kb haplotype that are Identical by Descent (IBD) in D*V individuals. Genotype data is from the 60K SNP chip and color coded according to genotype with missing data shown in black. SNP names in the IBD region are marked in red. A single SNP GGaluGA142157 at 38,806,246 bp (marked in yellow) is heterozygous in all D*V individuals, suggestive of a duplication.
(TIF)

S2 Fig Comb morphology of Wild-type (D*N), V-shaped (D*V) and Buttercup (D*C) chicken. Morphology of E12 and E18 (A, B) wild-type single comb (D*N), (C, D), V-shaped comb (D*V), and (E) E15 Buttercup comb (D*C). Note the reduced anterior part and the posterior split of the E18 V-shaped comb indicated by a black arrow. The nostril morphology is also affected. E; embryonic day, st; Hamburger & Hamilton developmental stage, n; nostril.
(TIF)

S1 Table Primer sequences. (DOCX)

## References

[1] Johnsson M, Gustafson I, Rubin C-J, Sahlqvist A-S, Jonsson KB, et al (2012) A sexual ornament in chickens is affected by pleiotropic alleles at HAO1 and BMP2, selected during domestication. PLoS Genet 8: e1002914 10.1371/journal.pgen.1002914 22956912 PMC3431302

[2] Schantz Von T, Tufvesson M, Göransson G (1995) Artificial selection for increased comb size and its effects on other sexual characters and viability in Gallus domesticus (the domestic chicken). Heredity 75: 518–529. 10.1038/hdy.1995.168

[3] Boije H, Harun-Or-Rashid M, Lee Y-J, Imsland F, Bruneau N, et al (2012) Sonic Hedgehog-signalling patterns the developing chicken comb as revealed by exploration of the pea-comb mutation. PLoS ONE 7: e50890 10.1371/journal.pone.0050890 23227218 PMC3515514

[4] Imsland F, Feng C, Boije H, Bed'hom B, Fillon V, et al (2012) The Rose-comb mutation in chickens constitutes a structural rearrangement causing both altered comb morphology and defective sperm motility. PLoS Genet 8: e1002775 10.1371/journal.pgen.1002775 22761584 PMC3386170

[5] Wright D, Boije H, Meadows JRS, Bed'hom B, Gourichon D, et al (2009) Copy number variation in intron 1 of SOX5 causes the Pea-comb phenotype in chickens. PLoS Genet 5: e1000512 10.1371/journal.pgen.1000512 19521496 PMC2685452

[6] Somes RG (1991) Some observations on high cavernous nostrils in the chicken. J Hered 82: 172–174. 2013692 10.1093/oxfordjournals.jhered.a111055

[7] Aldrovandi U (1600) Ornithologiae tomus alter cum indice copiosissimo. Bologna.

[8] Somes RG (1991) Duplex comb in the chicken: a multi-allelic trait. J Hered 82: 169–172. 2013691 10.1093/oxfordjournals.jhered.a111054

[9] Showell C, Binder O, Conlon FL (2004) T-box genes in early embryogenesis. Dev Dyn 229: 201–218. 10.1002/dvdy.10480 14699590 PMC1635809

[10] Dorshorst B, Okimoto R, Ashwell C (2010) Genomic regions associated with dermal hyperpigmentation, polydactyly and other morphological traits in the Silkie chicken. J. Hered 101: 339–350. 10.1093/jhered/esp120 20064842

[11] Groenen MAM, Megens H-J, Zare Y, Warren WC, Hillier LW, et al (2011) The development and characterization of a 60K SNP chip for chicken. BMC Genomics 12: 274 10.1186/1471-2164-12-274 21627800 PMC3117858

[12] Wragg D, Mwacharo JM, Alcalde JA, Hocking PM, Hanotte O (2012) Analysis of genome-wide structure, diversity and fine mapping of Mendelian traits in traditional and village chickens. Heredity 109: 6–18. 10.1038/hdy.2012.9 22395157 PMC3375411

[13] Rubin C-J, Zody MC, Eriksson J, Meadows JRS, Sherwood E, et al (2010) Whole-genome resequencing reveals loci under selection during chicken domestication. Nature 464: 587–591. 10.1038/nature08832 20220755

[14] Wong GK-S, Liu B, Wang J, Zhang Y, Yang X, et al (2004) A genetic variation map for chicken with 2.8 million single-nucleotide polymorphisms. Nature 432: 717–722. 10.1038/nature03156 15592405 PMC2263125

[15] Cooper GM (2005) Distribution and intensity of constraint in mammalian genomic sequence. Genome Res 15: 901–913. 10.1101/gr.3577405 15965027 PMC1172034

[16] Hutt FB, Mueller CD (1943) The linkage of polydactyly with multiple spurs and duplex comb in the fowl. Am Nat.77: 70–78. 10.2307/2457381

[17] Pitel F, Bergé R, Coquerelle G, Crooijmans R, Groenen MA, et al (2000) Mapping the naked neck (NA) and polydactyly (PO) mutants of the chicken with microsatellite molecular markers. Genet Sel Evol 32: 73–86. 10.1051/gse:2000107 14736408 PMC2706862

[18] Dunn IC, Paton IR, Clelland AK, Sebastian S, Johnson EJ, et al (2011) The chicken polydactyly (Po) locus causes allelic imbalance and ectopic expression of Shh during limb development. Dev Dyn 240: 1163–1172. 10.1002/dvdy.22623 21465618

[19] Maas SA, Fallon J (2005) Single base pair change in the long-range Sonic hedgehog limb-specific enhancer is a genetic basis for preaxial polydactyly. Dev Dyn 232: 345–348. 10.1002/dvdy.20254 15637698

[20] Bateson W, Punnett R (1908) Experimental studies in the physiology of heredity. Repts Evol Comm Roy Soc 4: 18–35.

[21] Pernaute B, Cañon S, Crespo M, Fernandez-Tresguerres B, Rayon T, et al (2010) Comparison of extraembryonic expression of Eomes and Cdx2 in pregastrulation chick and mouse embryo unveils regulatory changes along evolution. Dev Dyn 239: 620–629. 10.1002/dvdy.22176 20014105

[22] Marcucio RS, Cordero DR, Hu D, Helms JA (2005) Molecular interactions coordinating the development of the forebrain and face. Dev Biol 284: 48–61. 10.1016/j.ydbio.2005.04.030 15979605

[23] Hastings PJ, Lupski JR, Rosenberg SM, Ira G (2009) Mechanisms of change in gene copy number. Nat Rev Genet 10: 551–564. 10.1038/nrg2593 19597530 PMC2864001

[24] Andersson L (2013) Molecular consequences of animal breeding. Curr Opin Genet Dev 23: 295–301. 10.1016/j.gde.2013.02.014 23601626

[25] Dorshorst B, Molin A-M, Rubin C-J, Johansson AM, Strömstedt L, et al (2011) A complex genomic rearrangement involving the endothelin 3 locus causes dermal hyperpigmentation in the chicken. PLoS Genet 7: e1002412 10.1371/journal.pgen.1002412 22216010 PMC3245302

[26] Gunnarsson U, Kerje S, Bed'hom B, Sahlqvist A-S, Ekwall O, et al (2011) The Dark brown plumage color in chickens is caused by an 8.3-kb deletion upstream of SOX10. Pigment Cell Melanoma Res 24: 268–274. 10.1111/j.1755-148X.2011.00825.x 21210960

[27] Rubin C-J, Megens H-J, Martinez Barrio A, Maqbool K, Sayyab S, et al (2012) Strong signatures of selection in the domestic pig genome. Proc Natl Acad Sci 109: 19529–19536. 10.1073/pnas.1217149109 23151514 PMC3511700

[28] Sundström E, Komisarczuk AZ, Jiang L, Golovko A, Navratilova P, et al (2011) Identification of a melanocyte-specific, microphthalmia-associated transcription factor-dependent regulatory element in the intronic duplication causing hair greying and melanoma in horses. Pigment Cell Melanoma Res 25: 28–36. 10.1111/j.1755-148X.2011.00902.x 21883983

[29] Durkin K, Coppieters W, Drögemüller C, Ahariz N, Cambisano N, et al (2012) Serial translocation by means of circular intermediates underlies colour sidedness in cattle. Nature 482: 81–84. 10.1038/nature10757 22297974

[30] Allais-Bonnet A, Grohs C, Medugorac I, Krebs S, Djari A, et al (2013) Novel insights into the bovine polled phenotype and horn ontogenesis in Bovidae. PLoS ONE 8:e63512 10.1371/journal.pone.0063512.s007 23717440 PMC3661542

[31] Medugorac I, Seichter D, Graf A, Russ I, Blum H, et al (2012) Bovine polledness—an autosomal dominant trait with allelic heterogeneity. PLoS ONE 7:e39477 10.1371/journal.pone.0039477 22737241 PMC3380827

[32] Rothammer S, Capitan A, Mullaart E, Seichter D, Russ I, et al (2014) The 80-kb DNA duplication on BTA1 is the only remaining candidate mutation for the polled phenotype of Friesian origin. Genet Sel Evol 46: 44 10.1186/1297-9686-46-44 24993890 PMC4099407

[33] Kijas JM, Moller M, Plastow GS, Andersson L (2001) A frameshift mutation in MC1R and a high frequency of somatic reversions cause black spotting in pigs. Genetics 158: 779–785. 11404341 10.1093/genetics/158.2.779 PMC1461691

[34] Livak K (2001) Analysis of Relative Gene Expression Data Using Real-Time Quantitative PCR and the 2−ΔΔCT Method. Methods 25: 402–408. 10.1006/meth.2001.1262 11846609

[35] Li H, Durbin R (2009) Fast and accurate short read alignment with Burrows-Wheeler transform. Bioinformatics 25: 1754–1760. 10.1093/bioinformatics/btp324 19451168 PMC2705234

[36] DePristo MA, Banks E, Poplin R, Garimella KV, Maguire JR, et al (2011) A framework for variation discovery and genotyping using next-generation DNA sequencing data. Nat Genet 43: 491–498. 10.1038/ng.806 21478889 PMC3083463

[37] Vandesompele J, De Preter K, Pattyn F, Poppe B, Van Roy N, et al (2002) Accurate normalization of real-time quantitative RT-PCR data by geometric averaging of multiple internal control genes. Genome Biol 3:research0034 10.1186/gb-2002-3-7-research0034 12184808 PMC126239

[38] Hillel J, Groenen MAM, Tixier-Boichard M, Korol AB, David L, et al (2003) Biodiversity of 52 chicken populations assessed by microsatellite typing of DNA pools. Genet Sel Evol 35: 533–557. 10.1051/gse:2003038 12939204 PMC2697980

