# Supplementary figures and images for: A Genomic Duplication is Associated with Ectopic Eomesodermin Expression in the Embryonic Chicken Comb and Two Duplex-comb Phenotypes
Source: PLoS Genet. 2015 Mar 19;11(3):e1004947. doi: 10.1371/journal.pgen.1004947 (PMC4366209; doi:10.1371/journal.pgen.1004947)

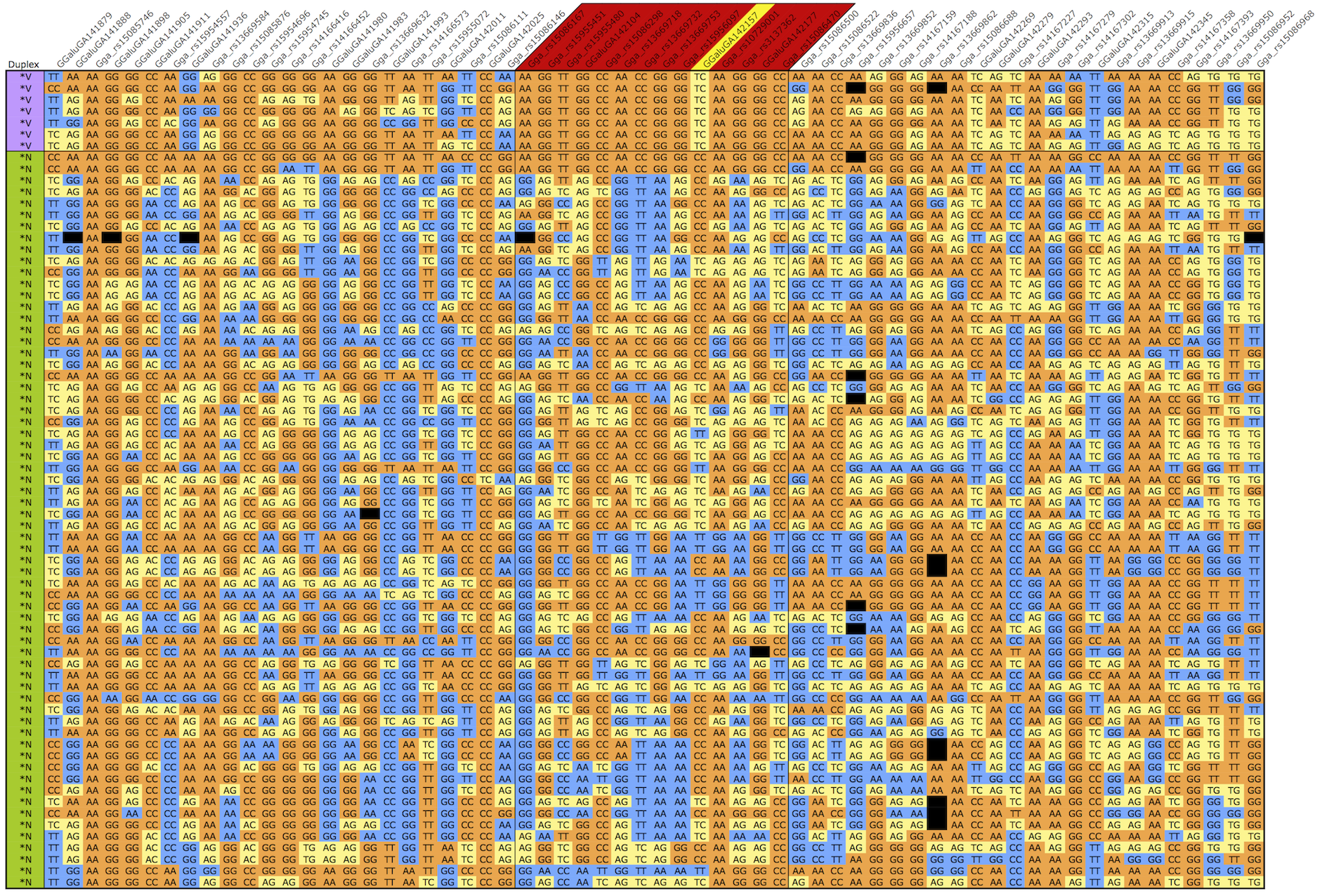

Supplement: S1 Fig — Genotype data is from the 60K SNP chip and color coded according to genotype with missing data shown in black. SNP names in the IBD region are marked in red. A single SNP GGaluGA142157 at 38,806,246 bp (marked in yellow) is heterozygous in all D*V individuals, suggestive of a duplication. (TIF) [file pgen.1004947.s001.tif]

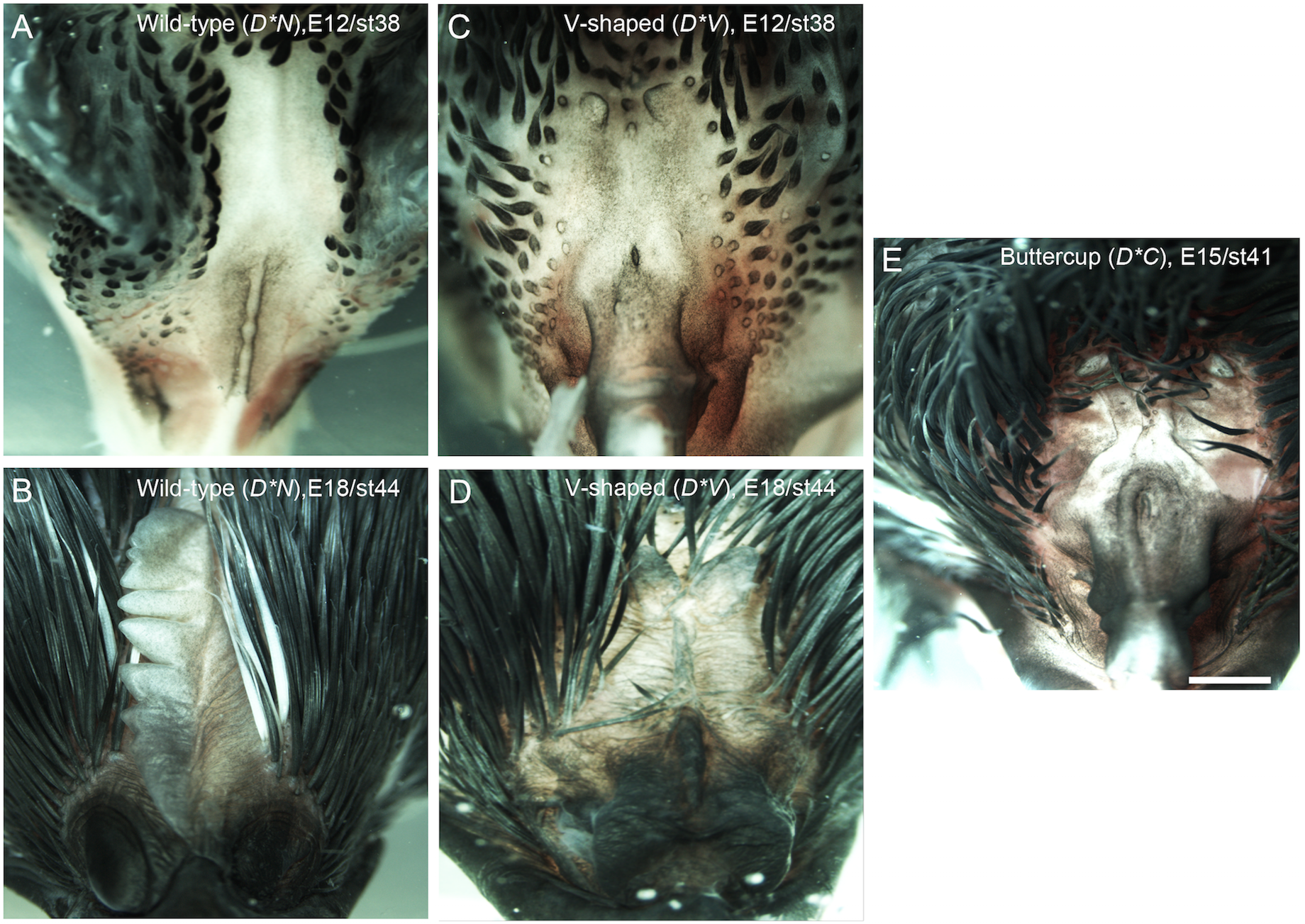

Supplement: S2 Fig — Morphology of E12 and E18 (A, B) wild-type single comb (D*N), (C, D), V-shaped comb (D*V), and (E) E15 Buttercup comb (D*C). Note the reduced anterior part and the posterior split of the E18 V-shaped comb indicated by a black arrow. The nostril morphology is also affected. E; embryonic day, st; Hamburger & Hamilton developmental stage, n; nostril. (TIF) [file pgen.1004947.s002.tif]
