# Supplementary material for: A Genomic Duplication is Associated with Ectopic Eomesodermin Expression in the Embryonic Chicken Comb and Two Duplex-comb Phenotypes
Source: PLoS Genet. 2015 Mar 19;11(3):e1004947. doi: 10.1371/journal.pgen.1004947 (PMC4366209; doi:10.1371/journal.pgen.1004947)
Supplement: S1 Table — (DOCX) [file pgen.1004947.s003.docx]

**Table S1.** Primer sequences.

| **Purpose** | **Primer Name** | **Primer Sequence** | **Chr.** | **Location (bp)** |
| --- | --- | --- | --- | --- |
| Genomic CNV | D_CNV_F | TGCCCAGAAAGCCTTACAC | 2 | 38,806,358 |
| Genomic CNV | D_CNV_P | 6FAM-TGAGAGGCTTCCACAGCTGACA-MGBNFQ | 2 | 38,806,397 |
| Genomic CNV | D_CNV_R | TGAGGGCAGAGGATTTACAA | 2 | 38,806,485 |
| Genomic CNV | SOX5_CNV_F | CCGTCATCCAGAGCACTTAC | 1 | 68,335,770 |
| Genomic CNV | SOX5_CNV_P | VIC-TCAAAGGCGAGGAGCCCC-TAMRA | 1 | 68,335,794 |
| Genomic CNV | SOX5_CNV_R | GGTCATCCTCTTCCTCATCATA | 1 | 68,335,886 |
| Diagnostic Test | D_5'_F | CAATTGCTTTGCAGTTTTGGATT | 2 | 38,798,386 |
| Diagnostic Test | D_5'_R | GTTCTGTTTTCCACTGCTGCAC | 2 | 38,798,653 |
| Diagnostic Test | D_3'_F | ATCCCTTTGCTACCACCACTGT | 2 | 38,818,264 |
| qPCR | EOMES_F | TTTCCCCGAAACGCAGTTC | 2 | 38,594,335 |
| qPCR | EOMES_R | TGTCTCTGAAGCCCTTTGCAA | 2 | 38,598,353 |
| qPCR | CMC1_F | TCCGCAAATAATGAGAGACCGA | 2 | 38,757,713 |
| qPCR | CMC1_R | TGCTGTGTTCTCTTCCCGACA | 2 | 38,822,064 |
| qPCR | AZI2_F | GCGCTAGTCACCGCATATGAA | 2 | 38,840,733 |
| qPCR | AZI2_R | CTGAACTGCATTCCTCCTCCAG | 2 | 38,844,753 |
| qPCR | β-actin_F | AGGTCATCACCATTGGCAATG | 6 | 18,777,232 |
| qPCR | β-actin_R | CCCAAGAAAGATGGCTGGAA | 6 | 18,781,252 |
